# The Removal of Platinum Group Metals, Cs, Se, and Te from Nuclear Waste Glass Using Liquid Sb Extraction and Phase Separation Methods

**DOI:** 10.3390/ma13225305

**Published:** 2020-11-23

**Authors:** Meng Zhang, Ying Lv, Zhanglian Xu, Sheng Wang, Jie Wang

**Affiliations:** 1 Shaanxi Key Laboratory of Advanced Nuclear Energy and Technology, and Shaanxi Engineering Research Center of Advanced Nuclear Energy, School of Nuclear Science and Technology, Xi’an Jiaotong University, No. 28, Xianning West Road, Xi’an 710049, Shaanxi, China; zm847203245@stu.xjtu.edu.cn; 2 College of Materials Science and Engineering, Xi’an Shiyou University, No. 18, 2nd East Dianzi Road, Xi’an 710065, Shaanxi, China; 180305@xsyu.edu.cn

**Keywords:** radionuclides, borosilicate glass, antimony, phase separation, recovery

## Abstract

Recovery of platinum group metals (PGMs: Pd, Ru, Rh), Cs, Se, and Te from molten borosilicate glass containing simulated high level radwaste through the combination of liquid metal extraction and phase separation method under reductive heat-treatment was studied. In this process, the PGMs were extracted in recovered liquid metal phase, where Sb and Bi metals were used as the collecting metals. Meanwhile, Cs, Se, and Te were enriched in the phase separated potassium-rich materials on glass surface, which were extracted by water. The type of liquid metals had profound influence on the extraction behaviors of PGMs and other fission products from the glass melt. As a result, except the near extraction efficiency of Pd, Sb showed higher affinity for Ru and Rh than Bi metal. The higher phase separation efficiency of potassium-rich materials led to the higher extraction efficiencies of Cs, Se, and Te in liquid Sb extraction than Bi. Among the examined conditions, using liquid Sb extraction, the Pd, Ru, and Rh extraction efficiencies were 78.6%, 62.1% and 100% in liquid Sb metal phase, and 93.76% of Cs, 60.4% of Se, and 23.65% of Te in leachate were obtained.

## 1. Introduction

The high-level radioactive liquid waste (HLLW) generated from spent fuels in the reprocessing facilities is immobilized into the borosilicate glass by using the Joule heated ceramic melter (JHCM) because of its high thermal efficiency and safety [1,2]. However, the existence of platinum group metals (PGMs) in the radwaste limited the advantages of the JHCM method for HLLW vitrification. These PGMs partially settled at the bottom of the melter during the vitrification process of HLLW due to their extremely low solubility (<1 wt %) in glass [3,4]. The formation of conductive metallic slag can cause severe loss of current in the glass [2], which may erode the melter and shorten its service life. In the JHCM, the accumulation of PGMs was addressed by reducing the bottom temperature to slow down the settling rate of PGMs [5] or redesigning the bottom drain to improve the discharge performance of PGMs [6]. However, these operations may become unavailable in the future when the ratio of PGMs to HLLWs increases with increasing fuel combustion. In other vitrification methods of HLLW, even if PGMs may not affect the melting process, they may pose problems and consequently devitrify the glass during the storage due to their low solubility in these existing high-level vitrified wastes (HLVWs). The result may lead to the leakage of the radionuclides. It should be noted that it is difficult to recover the PGMs from the existed vitrified glasses that have been encapsulated into stainless steel tanks. However, the development of the technique for efficient removal of PGMs prior to final vitrification of the HLLW that are present and generated in the future is necessary.

The liquid metal extraction has proven to be an efficient method to recover PGMs from ceramics and glasses. These PGMs can be extracted by alloying with liquid metals in the melting process. The first example using this method was mainly to recover the noble metals from exhausted automotive catalysts [7,8,9]. Burnham and coauthors [7] used common copper or iron as collecting metals to recycle cocatalysts Pd and Pt in a plasma arc furnace. Taninouchi and the coauthors [9] recovered PGMs from spent catalysts using Ni metal for magnetic separation. Inspired by this, Naito et al. [10,11] extracted PGMs in liquid metal lead from the simulated insoluble residue produced in high-level liquid waste. Uruga et al. [12,13] reported liquid metal Cu as the collecting metal for the recovery of PGMs and Mo using Si as the reducing agent. In this work, the removal of PGMs (Pd, Ru, Rh) from the simulated HLVWs was investigated by liquid Sb and Bi extraction. Sb and Bi metals were selected as the collecting metals because their metal oxides are easily reduced to the corresponding metals, all of which have much lower melting points than glass (273.1 °C for Bi, 630.7 °C for Sb and 820 °C for borosilicate glass), favoring the alloying with PGMs. On the other hand, it is worth noting that the simulated glasses used by Uruga and Naito et al. generally contained only few simulated fission products for the proof-to-concept test. However, in the actual HLVWs, the chemical compositions are more varied compared to the above-mentioned simulated glass. To verify this liquid metal extraction proposed in this study, such as a multicomponent glass as the HLVWs should be examined. In addition, in these existing HLVWs, besides ^107^Pd with half-life of 6.5 × 10^5^ y in PGMs, other long-lived fission products (LLFPs) with high level radioactivity and long half-lives, such as ^135^Cs, ^79^Se and ^128^Te [14,15], were contained and they should also be separated and converted into short-lived or stable nuclides through transmutation in the future. The extracted PGMs and ^137^Cs from the spent nuclear wastes are expected for the potential applications in pharmaceutical industry, petroleum, electronic devices and nuclear medicine industries [16,17,18]. Therefore, we tried to develop a recovery technique that can simultaneously extract PGMs and Cs, Se, and Te from the molten HLVWs based on the above-mentioned liquid metal extraction method.

In our previous studies, we have demonstrated the phase separation of Cs occurred on the surface of simulated lead soda-potash-borosilicate glass containing Cs, which was heat-treated under CO-rich atmosphere [19]. The reductive atmosphere was responsible for the formation of soda-potash-rich materials on the surface of the heat-treated glass, which favored the enrichment of Cs in this soda-potash-rich phase. Therefore, combining the above liquid metal extraction and the phase separation methods, in this study, a highly simulated HLVW that is similar to the actual HLVWs in compositions was utilized for simultaneous PGMs and Cs, Se, Te extraction tests by liquid metal Sb and Bi under the reductive heat treatment. In this process, the highly simulated HLVW, antimony oxide or bismuth oxide, potassium carbonate and carbon powder were mixed and melted at 1000 °C in a sealed crucible. During the reductive heat treatment, the antimony oxide or bismuth oxide were reduced to the corresponding metallic states by carbon powder, and PGMs were alloyed with the phase-separated liquid metal agglomerate. From the recovered metallic phases, the distributions of these PGMs in metal phases were demonstrated and the different affinities of PGMs from the glass melt by these two recovered metals were determined. Then, the extraction efficiencies of PGMs in these two recovered metals were compared. Meanwhile, the molten glass was further annealed at 700 °C to form the potassium-rich material phase on the surface of the glass in contact with the gas phase [19]. From the heat-treated contact surfaces of the glass phase, the phase separation efficiencies of potassium-rich materials and the extraction efficiencies of Cs, Se, and Te concentrated in this potassium-rich phase were compared. Finally, a flow chart of separation of simulated LLFPs prior to the vitrification process of HLLW and from the existing HLVWs was proposed by using the liquid Sb extraction.

## 2. Materials and Methods

### 2.1. The Preparation of the Simulated HLVW

The glass forming reagents (Na_2_CO_3_, H_3_BO_3_, SiO_2_, CaCO_3_, Al_2_O_3_, ZnO, Li_2_CO_3_) and several metal oxides and carbonates (La_2_O_3_, Nd_2_O_3_, ZrO_2_, Cs_2_CO_3_, PdO, Rh_2_O_3_, RuO_2_ and SeO_2_ and so on) were used to prepare the simulated HLVWs. La and Pd, Ru, Rh, and Cs were the representative simulated nuclides of lanthanides, PGMs, and alkali earth elements, respectively. These non-radioactive reagents were purchased from Wako Chemical Co. Ltd., Osaka, Japan. Certain amounts of the above reagents were ground and mixed in a 100 mL alumina crucible, which was placed into the furnace and heated at 1300 °C for 2 h. After the melting process, the melt was then naturally cooled to room temperature and ball-milled into powders for use. The components of the simulated HLVWs were measured using the method provided by Sawada [20]: The concentrations of the element were determined by the inductively coupled plasma mass spectrometry on a Perkin Elmer Optima 5300 DV (ICP-MS, Perkin Elmer, Inc., Hopkinton, MA, USA) after digesting the glass sample by a HF corrosion method for the detection of most of elements and by an alkali fusion method using Na_2_CO_3_ for the detection of Si and B, and using KOH and KNO_3_ for Ru detection. Table 1 summarized the compositions of the simulated HLVW. The fractions of PdO, Rh_2_O_3_, RuO_2_, Cs_2_O, SeO_2_ and TeO_2_ were 0.36 ± 0.04 wt %, 0.65 ± 0.09 wt %, 0.18 ± 0.02 wt %, 0.75 ± 0.05 wt %, 0.12 ± 0.02 wt % and 0.13 ± 0.01 wt %, respectively.

### 2.2. Reductive Heat Treatment

The liquid Sb extraction experiment was carried out under the reductive heat treatment as following: the simulated HLVW, Sb_2_O_3_ (particle size < 80 mesh, used as the scavenging agent), K_2_CO_3_ (particle size < 100 mesh, used as a flux) and the activated carbons (particle size < 80 mesh, used as the reductant) were added in a 30 mL alumina crucible. Then the 30 mL alumina crucible with an alumina cover was put into a larger alumina crucible (100 mL) containing Al_2_O_3_ powder. The 100 mL crucible was also covered by an alumina cover and then transferred in an electric furnace. The temperature was raised to 1000 °C over 30 min and then maintained for 1 h. After that, the temperature was reduced to 700 °C for annealing the melt for 2 h. Finally, the glass sample was cooled naturally to room temperature. The obtained glass was called “heat-treated product”. The metal button was completely recovered by crushing the bottom of the crucible using hammer. The liquid Bi extraction was performed using Bi_2_O_3_ as the scavenging agent with other above conditions unchanged. Table 2 presents the amounts of the compounds used in the experiment. Each batch was repeated twice.

### 2.3. The Elution Test

The elution test was carried out on the obtained heat-treated products. The 30 mL alumina crucible containing the heat-treated product was placed in a large beaker (200 mL) and then 150 mL of deionized water was added into the beaker. A mechanical stir bar was inserted on top of the solution and the solution was stirred at 300 rpm for 2 h at room temperature. After that, the suspended particles in the leachate were filtered using a 1 μm paper filter. The concentrations of K, Cs, Se, Pd, Rh, Ru, and Te in the leachate were measured by ICP-MS. The elution test was performed twice for the reproducibility.

The extraction efficiencies of the above elements can be determined by Equation (1).

Extraction efficiency on the elements in water = E_i_/M_i_ × 100 i = K, Cs, Se, Pd, Ru, Rh, Te(1)

where E_i_ represents the amount of the i element eluted from the heat-treated product using deionized water, and M_i_ represents the amount of the i element contained in the simulated HLVW.

### 2.4. The Extraction of PGMs from the Metal Phase

The metal buttons recovered from the heat-treated products were placed in a 100 mL beaker. Then 40 mL of the aqua regia (HCl:HNO_3_ = 3:1 v/v) was added and heated at 100 °C for 3 h to digest the metal buttons completely. After digestion, the aqua regia was collected by vacuum filtration using a 1 μm paper filter. The obtained solution was further diluted to 100 mL using HNO_3_. The concentrations of the elements in the obtained solution were determined by ICP-MS. The analysis was repeated twice. The extraction efficiencies of the above elements in the metal phase were determined by Equation (2):

Extraction efficiency of M in metal phase = A^M^/A_0_^M^ × 100 M = Pd, Ru, Rh, Cs, Te and Se(2)

where A^M^ represents the amount of M in the metal phase digested in aqua regia. A_0_^M^ represents the amount of M contained in the simulated HLVW.

### 2.5. Characterization

The morphology of the surfaces of the heat-treated products and the metal buttons was observed using field-emission scanning electron microscopy (FE-SEM, Seiko Instruments Co. Ltd., Chiba, Japan; Zeiss, Oberkochen, Germany). The mapping elements on the selected area were analyzed using energy-dispersive X-ray spectrometer (EDS, Bruker AXS Co. Ltd., Kanagawa, Japan). The phases of the sample were determined using X-ray powder diffraction (XRD, D8 ADVANC, Bruker, Germany) using a CuKα with a Ni filter (40 kV, 40 mA).

## 3. Results and Discussion

### 3.1. XRD Measurement of the Reduced Metal Phase

**Figure 1:**
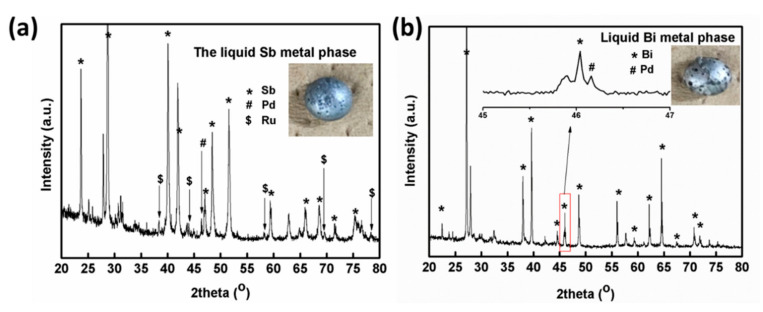
XRD patterns and the pictures (inset) of (**a**) the reduced Sb metal from the batch 1 and (**b**) the reduced Bi metal from the batch 2 after heat treatment under reducing atmosphere.

In this work, Sb_2_O_3_ and Bi_2_O_3_ were used as the scavenging agents for extracting PGMs from the simulated HLVWs using the reductive heat treatment. After the reductive heat treatment, the metallic buttons would be generated at the bottom of the crucible through the reduction by adding carbon powder. To clarify the phase of the reduced materials obtained, XRD measurements were carried out. Figure 1a displays XRD pattern of the reduced metal phase with a light gray metallic luster (inset in Figure 1a) from the batch 1. The main phase of the reduced metallic phase from the batch 1 is identified as Sb metallic substance (JCPDS No. 00-085-1322) [21]. The peaks at 38.3, 44.0, 58.3, 69.4, and 78.5° correspond to the diffractions of the (100), (101), (102), (110), and (103) planes of the hexagonal close-packed (hcp) Ru metal (JCPDS card No. 06-0663), respectively [22]. The peak centered at 46.4° corresponds to the (200) diffraction of the Pd substance [23]. The detection of Pd and Ru metals suggests that Pd and Ru can be extracted by liquid Sb metal from the simulated HLVWs. The characteristic peaks that belong to Rh metal are not detected which may be due to the low content in reduced metallic agglomerate. For comparison, Figure 1b presents the XRD pattern of the reduced metal phase with a brighter metallic luster (inset picture in Figure 1b) from the batch 2. These peaks are considered to be characteristic of the rhombohedral phased Bi metal (JCPDS No. 05-0519) [24], and a minor reflection of Pd metallic phase (inset spectrum in Figure 1b) in the bismuth button is also observed. No Ru or Rh phase is observed in the reduced Bi agglomerate. This may be due to low extraction of Ru and Rh by the reduced Bi metal. By contrast, the results demonstrate that liquid Sb and Bi metal have different extraction capacity to PGMs from the simulated HLVWs. Noting that 70.6 wt % of Sb and 62 wt % of Bi were recovered, respectively. Higher recovery efficiency of Sb metal was obtained than Bi metal based on the present conditions.

### 3.2. PGMs Extraction in Reduced Metallic Buttons

**Figure 2:**
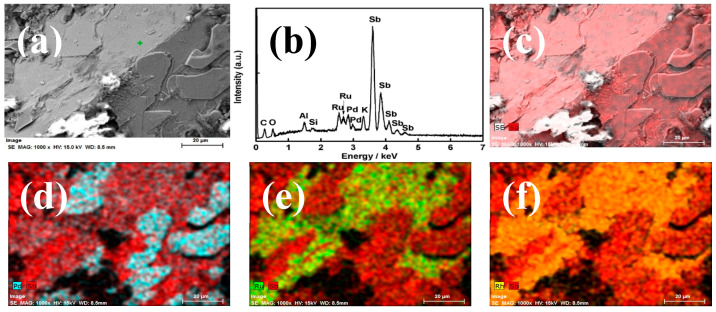
(**a**) FE-SEM images, (**b**) EDS analysis for the Sb metal obtained from the batch 1 and the corresponding elemental mapping: (**c**) Sb; (**d**) Pd and Sb; (**e**) Ru and Sb; (**f**) Rh and Sb.

**Figure 3:**
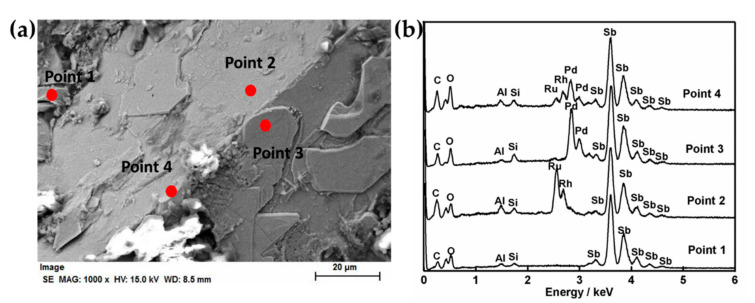
(**a**) FE-SEM image and (**b**) EDS spectra of point analysis on the recovered Sb metal phase-separated from the heat-treated product in batch 1.

The distribution of the extracted PGMs (Pd, Ru and Rh) in the reduced liquid metals obtained from the simulated HLVWs after the reductive heat-treatment was further revealed by FE-SEM and EDS analysis. Figure 2 presents the morphology of the surface of Sb metal, corresponding EDS results of Sb and PGMs in Sb metallic phase. The FE-SEM image in Figure 2a shows the flat surface of the recovered Sb metal, which was layered with many sheets on the surface. The EDS examination of the whole observation area (Figure 2b) demonstrated the presence of Sb, Pd, Ru, and Rh with strong signals, suggesting PGMs were successfully extracted into the reduced Sb button. Besides PGMs, the presence of K, Si, Al, C and O may be attributed to the attached glass oxide (the white materials in Figure 2a) on the surface of Sb metal. The corresponding elemental mapping of Sb and PGMs were displayed in Figure 2c–f. It can be seen that the observed area is composed of metallic Sb (Figure 2c) and the attached sheets mainly consist of Pd (Figure 2d); The Ru in the button is observed to be segregated in a separate phase (Figure 2e), where some Rh (Figure 2f) and an insignificant amount of Pd were also contained. The detailed compositions in the above different areas were further evidenced by the point analysis using EDS, as shown in Figure 3. The area at point 1 shows only the presence of Sb metal and the attached sheets are mainly composed of Pd and Sb (point 3 in Figure 3). Segregation of Ru and Rh-rich phase was observed in point 2, and point 4 shows the coexistence of Sb, Pd, Rh and Ru in Figure 3. The extracted PGMs were not alloyed uniformly, showing a separated phase in Sb metal.

**Figure 4:**
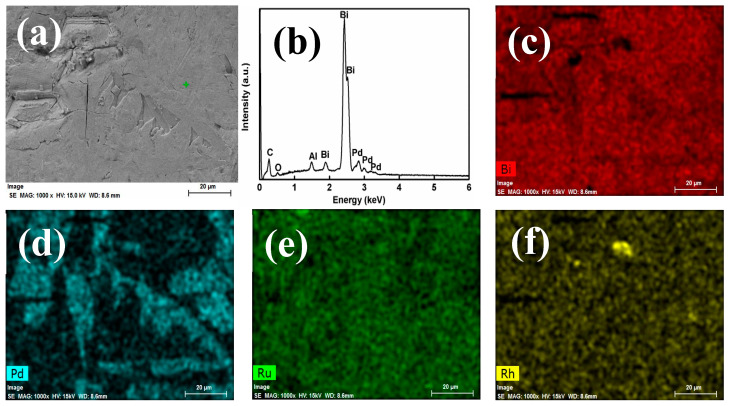
(**a**) FE-SEM image, (**b**) EDS analysis for the recovered liquid Bi metal from the batch 2 and the corresponding elemental mapping: (**c**) Bi; (**d**) Pd; (**e**) Ru; (**f**) Rh.

**Figure 5:**
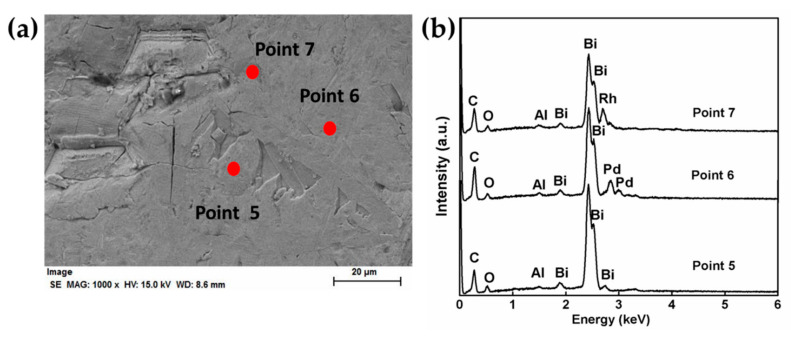
(**a**) FE-SEM image and (**b**) EDS spectra of point analysis on the recovered Bi metal phase separated from the heat-treated borosilicate glass in batch 2.

Figure 4 shows the morphology of the surface of metallic Bi button and the corresponding EDS results of Bi and PGMs. Unlike Sb metal, the surface of Bi metal showed relatively flat (Figure 4a). The only presence of Bi, Pd, Al, O and C was observed in whole observed area. No peaks that belong to Ru and Rh were detected. The corresponding elemental mapping images of Bi and PGMs were demonstrated in Figure 4c–f. The whole observed area is composed of Bi metal (Figure 4c) and many separated Pd-rich areas were distributed along with Bi metal (Figure 4d). The Ru (Figure 4e) and Rh (Figure 4f) present here seemed to alloy with Bi metal uniformly, which was different from the separated alloy in Sb metal. Only few concentrated areas of Rh were observed. The point analysis in the above varied areas was further performed, as shown in Figure 5. Most of the areas are rich in Bi in point 5 of Figure 5. Segregation of Pd-rich phase was observed in point 6, while point 7 confirms the Rh. However, the detection of Ru was not observed in all observed areas. This may be ascribed to the low concentration of Ru in the reduced Bi metal or the uniform distribution in Bi metal phase. Although EDS is a semi quantitative analysis and not sufficient for a quantitative analysis, the obtained data are still useful to indicate the extraction behaviors to PGMs by the liquid metals from the simulated HLVWs. Based on the observation results from EDS analysis, different distributions of PGMs, especially Ru and Rh were confirmed in the recovered metal Sb and metal Bi phase.

The extraction efficiencies of PGMs in reduced Sb button and Bi button separated from the heat-treated HLVWs were compared and the results are provided in Table 3. In batch 1, approximately 80% of the Pd was extracted into the Sb metal button, and the Bi metal extracted ca.88% of the Pd from the batch 2, both showing high extraction fraction of the Pd in liquid metals. While the extraction efficiencies of Rh and Ru in the recovered Sb metal phase were much higher than those in Bi metal phase. Over 100% of Rh and 62% of Ru were achieved in Sb metal phase. It is worth noting that Rh and Ru form borosilicate glass are difficult to be extracted even using strong acid because of the stability and durability of the nuclear waste glass [25] and it turns out that our approach is very effective to extract these PGMs using liquid Sb extraction. By contrast, only 42.1% of Rh and 0.53% of Ru were extracted into Bi metal phase. The Sb metal showed significantly different ability for extracting Ru and Rh to the Bi metal. In particularly, the Bi metal almost did not extract Ru. Compared to the high extraction efficiencies of Pd and Rh in both recovered metallic buttons, the lower Ru recovery efficiency was attributed to the formation of RuO_4_, which is volatile during the melting process [26]. As shown in the above elemental mapping analysis, Ru and Rh were observed to be extracted into the same phase in the recovered Sb button. It has been also reported that about half the Ru forms a Ru-based alloy such as -Ru (Mo, Tc and Rh) during the intermediate storage of a HLLW [27]. It is thus deduced that the lower recovery of Rh in recovered Bi button may be correlated with nearly no extraction of Ru by liquid Bi metal. It was reported that the extraction of PGMs was independent on the types of scavenger oxides and a great plenty of the PGMs could be recovered as long as the scavenger can be reduced and form a collectible sample [28]. However, in our present study, even the scavenger oxides (Sb_2_O_3_ and Bi_2_O_3_) were both able to be reduced to the collectible buttons, the differences in the extraction behavior of PGMs in the recovered Sb button and Bi button were determined, showing the different affinity of the collecting metals for PGMs.

The extraction fraction of other fission products (Cs, Se and Te) was also examined in the recovered Sb button and Bi button. As shown in Table 3, the concentrations of Cs, Se and Te extracted into the metal phase are much less than PGMs (except Ru in recovered Bi button). This may be due to the fact that the oxides of PGMs have a higher free energy of formation (relatively small negative values) compared to the oxides of other elements, as seen from the Ellingham oxide diagram. The oxides of Cs, Se and Te are more stable, thereby they remained in the glass phase. According to the above results, it is deduced that Sb can be used to remove PGMs from HLLW prior to the final vitrification of HLLW to borosilicate glass by the JHCM method.

### 3.3. The Extraction of Other Fission Products from the Heat-Treated Glass Phase

**Figure 6:**
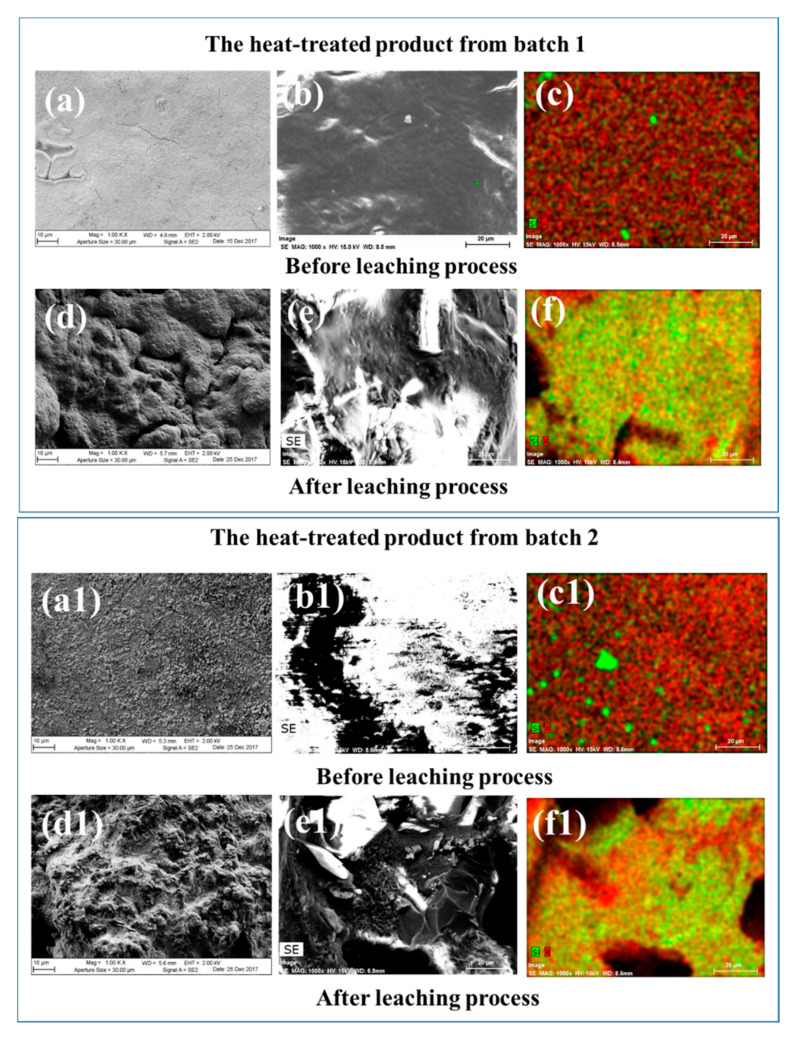
The surface morphology of the heat-treated products obtained from the two batches: FE-SEM images of the glass surface batch 1 and their corresponding mapping images of silicon and potassium elements before (**a**–**c**) and after (**d**–**f**) leaching process; FE-SEM images of the glass surface batch 2 and their corresponding mapping images of silicon and potassium elements before (**a1**–**c1**) and after (**d1**–**f1**) leaching process.

**Figure 7:**
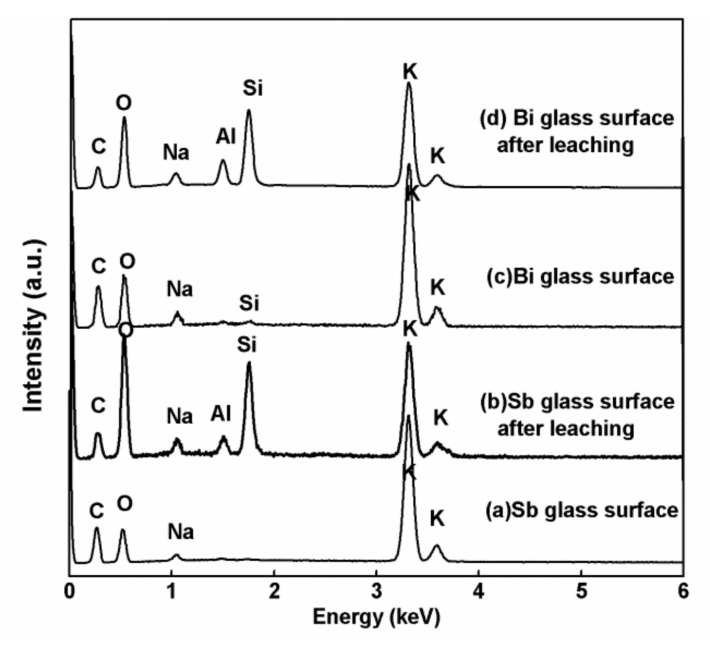
EDS spectra of the entire observed surfaces of the heat-treated products obtained from batch 1 and batch 2 before and after leaching process.

As mentioned in the above section, besides PGMs, the existing HLVWs contained other radioactive nuclides such as Cs, Se and Te with long half-lives. From the view of storage management, they should also be extracted from the HLVW and converted into short-lived or stable nuclides by transmutation. The enrichment of Cs on the surface of the glass was previously observed in a simple Cs-containing lead soda-potash-borosilicate glass after the reductive heat treatment [19]. The cesium enrichment was related to the formation of soda-potash-rich materials due to the reaction of glass materials with CO gas on the surface. In this study, we inferred that the CO-containing atmosphere might be formed in the sealed reactor due to the insufficient combustion of carbon powders during the heat treatment. To confirm the phase separation of cesium and other fission products in this highly simulated HLVW with complex compositions, the phase separation of potassium from the glass was first investigated. Figure 6 shows the FE-SEM images of the contact surfaces of the heat-treated products obtained from batch 1 and batch 2 before and after leaching process and their corresponding mapping images of silicon and potassium elements. In batch 1 and batch 2, the morphologies of the obtained contact surfaces both showed relatively smooth (Figure 6a,b,a1,b1) and both became rather rough after leaching process (Figure 6d,e,d1,e1). In mapping images, potassium was phase separated from silicon on both the contact surfaces (Figure 6c,c1). After leaching treatment, the condensed potassium-rich phase on the contact surface disappeared in both types of the heat-treated products (Figure 6f,f1). These changes of contact surfaces were confirmed from the EDS spectra obtained from the entire observed area in Figure 7. The intensities of potassium on the contact surfaces decreased and the intensities of silicon significantly increased after the leaching treatment for both types of the heat-treated products (Figure 7b,d) compared with the untreated ones (Figure 7a,c). According to the results, phase separation of potassium-rich materials on the contact surface occurred in both types of the heat-treated products and the potassium-rich materials could be eluted by water. The phase separation efficiency of potassium on the contact surface was further demonstrated between the two types of heat-treated products. Table 4 shows the corresponding concentrations of potassium, silicon and sodium on the contact surfaces, determined by EDS results in Figure 7. Silicon and sodium elements belong to the compositions of the raw HLVWs. Thus, the mass ratio of potassium to silicon and sodium (K/(Si + Na)) was calculated from Table 4 and can be considered as an indicator of phase separation efficiency of potassium. As a result, the K/(Si + Na)) ratio for the heat-treated product from the batch 1 was 19.33, which was nearly twice larger than that from the batch 2 (K/(Si + Na) ratio = 10.37). We have previously demonstrated that alkali metal oxide enrichment was promoted by the efficiency of the reducing lead oxides in the glass [17]. It is thus that the higher phase separation efficiency of potassium-rich materials from the batch 1 may be attributed to the higher recovery efficiency of Sb_2_O_3_ than Bi_2_O_3_, as pointed out in Section 3.1. Therefore, the phase separation may proceed more effectively in the batch 1 using Sb_2_O_3_ as the scavenging agent. It is worth noting that the signals of Cs, Se and Te were not detected in EDS spectra in both cases (Figure 7). This may be due to the extremely low content in the highly simulated HLVWs.

To determine the leaching characteristics of Cs, Se, and Te in the heat-treated products, the extraction efficiencies of these elements in the leachate were quantitatively compared and provided in Table 5. The extraction efficiencies of Cs in the leachate were 93.76% for batch 1 and 73.51% for batch 2. Compared with the Cs extraction, the Se and Te extractions differed more significantly between these two batches. The extraction fractions of Se and Te from the heat-treated products were 60.4%, 23.65% for the batch 1 and 2.59%, 4.93% for the batch 2, respectively. One possible reason for this difference is that the phase separation efficiencies are different between the treatment of Sb-glass and Bi-glass. In addition, the extraction fractions of PGMs in the leachate were extremely low in both cases, as shown in Table 5.

### 3.4. The Proposed LLFPs Separation Process

**Figure 8:**
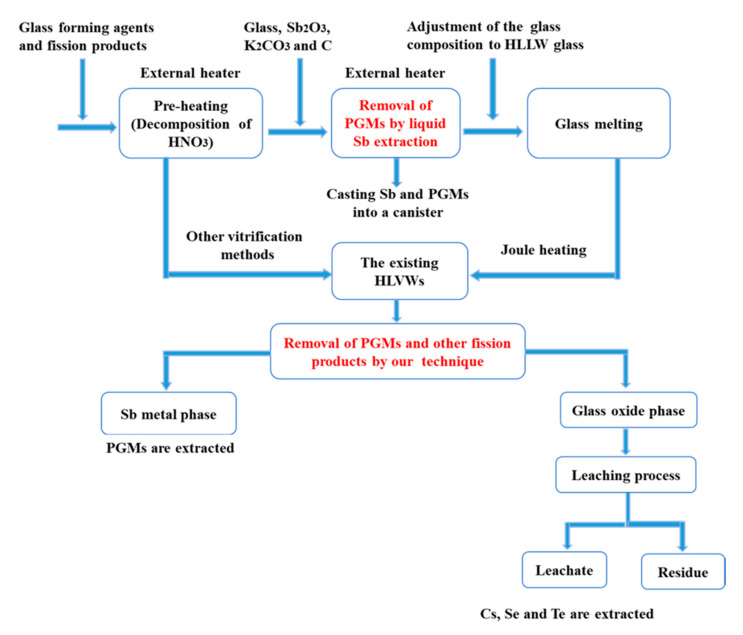
Flow chart of the separation of platinum group metals (PGMs) and other fission products by our proposed technique.

Based on the experimental results obtained here, the separation of PGMs and other fission products from the highly simulated HLVWs can be achieved by our proposed extraction method. This provided the possibility for the removal of PGMs prior to the vitrification process of HLLW in Joule heating method and also the possibility for the recovery of PGMs and other fission products from the existing HLVWs. As a summary, Figure 8 illustrates a flow chart of the separation of PGMs and other fission products by our proposed technique in the above processes. In Joule heating method, in order to decompose HNO_3_, glass forming agents and HNO_3_ solution containing fission products are fed into the melter for pre-heating treatment in the external heater. Then, the glass containing the scavenging agent (Sb_2_O_3_) and the reducing agent (C) are put into the melter. Then, the PGMs are alloyed with the liquid Sb metal phase. This liquid alloy metal phase is poured into a stainless steel canister from the bottom of the melter. After removing the PGMs from the molten glass, the composition of the glass was adjusted to meet the specifications of the HLVW. The glass is then melted using a Joule heating system to form the HLVWs. Together with the existing HLVWs generated by other vitrification methods, the PGMs and other fission products (Cs, Se and Te) can be recovered from these existing HLVWs by the liquid Sb extraction and phase separation methods. In the reduction-melting stage of these existing HLVWs, the PGMs can be extracted into the liquid Sb metal phase. In the next annealing stage, Cs, Se and Te will be phase separated in the potassium-rich materials on the contact surface. From the potassium-rich materials, Cs, Se and Te can be effectively extracted by leaching. Although the extraction efficiency of Te is still low, the proposed separation technique can be improved in the future and the study is under the way.

## 4. Conclusions

In summary, we present a technique combining liquid metal Sb extraction and phase separation to efficiently and simultaneously recover PGMs, Cs, Se and Te from highly simulated HLVWs under the reductive heat treatment. A higher extraction efficiency of PGMs and higher affinity for Ru and Rh were obtained from the recovered liquid Sb metal phases than liquid Bi metal phases. At the contact surface of the heat treatment products, the higher phase separation efficiency of potassium-rich material in Sb glass compared to Bi glass is responsible for the enrichment of Cs, Se and Te at the contact surface, leading to the higher extraction efficiency of Cs, Se and Te by leaching treatment. This study proposes a promising process to recover PGMs by using liquid Sb extraction and other fission products by using phase separation strategy as a pretreatment prior to Joule heating. This method is also expected to be used for the extraction of precious metals from the precious metal-containing glass in other industrial glass applications.

## Figures and Tables

**Table 1:** Chemical compositions of the simulated high-level vitrified wastes (HLVW).

	Concentration (wt %)	Error (%)
SiO_2_	50.5	1.3
B_2_O_3_	14.9	0.3
Na_2_O	10.24	0.5
CaO	3.08	0.9
Al_2_O_3_	5.47	1.1
ZnO	3.21	0.8
Li_2_O	3.06	0.7
RuO_2_	0.65	0.09
Rh_2_O_3_	0.18	0.02
PdO	0.36	0.04
SeO_2_	0.12	0.02
Cs_2_O	0.75	0.05
TeO_2_	0.13	0.01
ZrO_2_	1.31	0.1
MnO	0.34	0.04
BaO	0.54	0.03
La_2_O_3_	0.33	0.1
CeO_2_	1.94	0.4
Nd_2_O_3_	1.39	0.6
Gd_2_O_3_	1.29	0.8
Fe_2_O_3_	0.21	0.02

**Table 2:** The amounts of the compounds in batch heat treatment (unit: gram).

	**Simulated Glass**	**K_2_CO_3_**	**Sb_2_O_3_**	**Bi_2_O_3_**	**Carbon**
Batch 1	2.5	5	0.5	0	0.5
Batch 2	2.5	5	0	0.5	0.5

**Table 3:** The extraction efficiency of the elements in different liquid metal phase.

Elements	Mean Extraction Efficiency (wt %)	
	Sb Metal Phase	Bi Metal Phase
Pd	78.6	88.1
Rh	107	42.1
Ru	62.1	0.53
Se	N.D.	N.D.
Te	0.13	0.73
Cs	1.8	2.2

**Table 4:** Concentrations of K, Si and Na on the contact surfaces of the heat-treated products, determined by the entire EDS analysis in FE-SEM.

Elements	Concentration of Elements (wt %)			
	Heat-Treated Contact Surface from Batch 1	Heat-Treated Contact Surface from Batch 1 after Leaching	Heat-Treated Contact Surface from Batch 2	Heat-Treated Contact Surface from Batch 2 after Leaching
K	30.74	13.91	26.13	17.37
Si	N.D.	7.61	0.25	8.68
Na	1.59	2.1	2.27	1.29

**Table 5:** The extraction efficiency of the elements in different liquid metal phase.

Elements	Mean Extraction Efficiency (wt %)	
	Heat-Treated Contact Surface from Batch 1	Heat-Treated Contact Surface from Batch 2
Cs	93.76	73.56
Se	60.40	2.59
Te	23.65	4.93
Pd	N.D.	N.D.
Rh	0.22	0.14
Ru	<0.01	1.83

## References

[1] Chapman C.C., Pope J.M., Barnes S.M. (1986). Electric melting of nuclear waste glasses State of the art. J. Non-Cryst. Solids.

[2] Luckscheiter B., Nešović M. (1996). Development of glasses for the vitrification of high level liquid waste (HLLW) in a joule heated ceramic melter. Waste Manag..

[3] Akai T., Nishii J., Yamashita M., Yamanaka H. (1997). Chemical behavior of platinum-group metals in oxide glasses. J. Non-Cryst. Solids.

[4] Ojovan M.I., Lee W.E. (2005). An Introduction to Nuclear Waste Immobilisation.

[5] Jena H., Kutty K.V.G., Rao P.R.V. (2011). Effect of temperature on the extraction of Pd by liquid tin from molten borosilicate glass containing simulated radwaste. J. Non-Cryst. Solids.

[6] Igarashi H., Takahashi T. (1991). The draining of noble metals in vitrified nuclear waste by a melter with a sloping floor. Glass Technol..

[7] Burnham R.F., Harry J.E., Gibbon A. (1987). Plasma Arc Furnace. European Patent Application.

[8] Kumar S., Pavloudis T., Singh V., Nguyen H., Steinhauer S., Pursell C., Clemens B., Kioseoglou J., Grammatikopoulos P., Sowwan M. (2018). Hydrogen Flux through Size Selected Pd Nanoparticles into Underlying Mg Nanofilms. Adv. Energy Mater..

[9] Vardon D.R., Settle A.E., Vorotnikov V., Menart M.J., Eaton T.R., Unocic K.A., Steirer K.X., Wood K.N., Cleveland N.S., Moyer K.E. (2017). Ru-Sn/AC for the Aqueous-Phase Reduction of Succinic Acid to 1, 4-Butanediol under Continuous Process Conditions. ACS Catal..

[10] Garrett C.E., Prasad K. (2004). The Art of Meeting Palladium Specifications in Active Pharmaceutical Ingredients Produced by Pd-Catalyzed Reactions. Adv. Synth. Catal..

[11] Zhang P., Yang G., Tana L., Aia P., Yang R., Tsubakia N. (2018). Direct synthesis of liquefied petroleum gas from syngas over H-ZSM-5 enwrapped Pd-based zeolite capsule catalyst. Catal. Today.

[12] Benson M., Bennett C.R., Harry J.E., Patel M.K., Cross M. (2000). The recovery mechanism of platinum group metals from catalytic converters in spent automotive exhaust systems. Resour. Conserv. Recycl..

[13] Taninouchi Y., Watanabe T., Okabe T.H. (2017). Recovery of platinum group metals from spent catalysts using electroless nickel plating and magnetic separation. Mater. Trans..

[14] OECD-NEA (2007). Actinide and Fission Product Partitioning and Transmutation Status and Assessment Report.

[15] OECD-NEA (2003). Accelerator-Driven Systems (ADS) and Fast Reactors (FR) in Advanced Nuclear Fuel Cycles—A Comparative Study.

[16] Naito K., Matsui T., Tanaka Y. (1986). Recovery of noble metals from insoluble residue of spent fuel. J. Nucl. Sci. Technol..

[17] Naito K., Matsui T., Nakahira H., Kitagawa M., Okada H. (1991). Recovery and mutural separation of noble metals form the simulated insoluble residue of spent fuel. J. Nucl. Mater..

[18] Uruga K., Arita Y., Enokida Y., Yamamoto I. (2007). Removal of platinum group metals contained in molten glass using copper. J. Nucl. Sci. Technol..

[19] Xu Z., Okada T., Nishimura F., Yonezawa S. (2016). Phase separation of cesium from lead borosilicate glass by heat treatment under a reducing atmosphere. J. Hazard. Mater..

[20] Uruga K., Sawada K., Enokida Y., Yamamoto Y. (2008). Liquid metal extraction for removal of molybdenum from molten glass containing simulated nuclear waste elements. J. Nucl. Sci. Technol..

[21] Altin E., Oz E., Erdem M., Demirel S., Aydogdu Y., Altin S. (2015). Thermoelectric and mechanical properties of Mg-Al-Sb alloys. J. Mater. Sci. Mater. Electron..

[22] Liu J., Bai P., Zhao X.S. (2011). Ruthenium nanoparticles embedded in mesoporous carbon microfibers: Preparation, characterization and catalytic properties in the hydrogenation of D-glucose. Phys. Chem. Chem. Phys..

[23] Petla R.K., Vivekanandhan S., Misra M., Mohanty A.K., Satyanarayana N. (2012). Soybean (Glycine max) leaf extract based green synthesis of palladium nanoparticle. J. Biomater. Nanobiotechnol..

[24] Kumar P., Singh J., Pandey A.C. (2013). Rational low temperature synthesis and structural investigations of ultrathin bismuth nanosheets. RSC Adv..

[25] Takao K., Mori T., Kubo M., Uehara A., Ikeda Y. (2019). Wet chemical processing for nuclear waste glass to retrieve radionuclides. J. Hazard. Mater..

[26] Kamizono H., Kikkawa S., Togashi Y., Tashiro  S. (1989). Volatilization of 137Cs and 106Ru from borosilicate glass containing actual high-level waste. J. Am. Ceram. Soc..

[27] Kleykamp H. (1988). The chemical-state of fission-products in oxide fuels at different stages of the nuclear fuel cycle. Nucl. Technol..

[28] Jensen G., Platt A.M., Mellinger G.B., Bjorklund W.J. (1984). Recovery of noble metals from fission products. Nucl. Technol..

